# Enhancing Clinical Pharmacy Specialist Involvement in Transitions of Care Focusing on Ambulatory Care Sensitive Conditions within a Veterans Affairs Healthcare System

**DOI:** 10.3390/pharmacy8010047

**Published:** 2020-03-22

**Authors:** Morgan Fisher, Amber Cardoza, Autumn Gordon, Molly Howard, Lynsey Neighbors, Addison Ragan

**Affiliations:** Department of Pharmacy, Central Alabama Veterans Healthcare System, 215 Perry Hill Road, Montgomery, AL 36109, USA; Amber.Cardoza@va.gov (A.C.); Autumn.Gordon@va.gov (A.G.); Molly.Howard2@va.gov (M.H.); Lynsey.Neighbors@va.gov (L.N.); Addison.Holder@va.gov (A.R.)

**Keywords:** transitions of care, care transitions, medication management, readmissions, pharmacy, clinical pharmacy, ambulatory care

## Abstract

The purpose of this quality improvement project was to evaluate the impact of clinical pharmacy specialist (CPS) involvement in the post-discharge period on 30-day readmission rates within a Veterans Affairs Healthcare System. Patients eligible for inclusion were discharged from a Veterans Affairs (VA) acute care facility with a principle or secondary diagnosis of heart failure (HF), chronic obstructive pulmonary disease (COPD), or both HF and COPD from 15 October 2018 through 14 January 2019. CPSs functioning as a mid-level provider with a scope of practice conducted telephone and in-clinic medication management appointments within 7 and 21 days post-discharge for qualifying patients discharged with a principle or secondary diagnosis of HF or COPD. CPS appointments focused on medication reconciliation, ensuring continuity of care, disease state counseling, and medication management. By enhancing the role of the CPS in the post-discharge period, there was an observed decrease in 30-day COPD index (*p* = 0.35), HF index (*p* = 0.23), and all-cause (*p* = 0.62) readmission rates from pre- to post-intervention. The results of this intervention show that CPS intervention in the post-discharge period may reduce index and all-cause readmission rates for patients discharged with a principle or secondary discharge diagnosis of COPD or HF.

## 1. Introduction

Ineffective care transitions, also known as transitions of care (TOC), lead to adverse events and higher hospital readmission rates and therefore, have a detrimental effect on quality of life, morbidity, and mortality for patients [1,2,3,4]. In addition, the rate of hospital readmission shortly after discharge may be an indicator of quality of care transitions and 30-day readmission rates have become standard to measure effectiveness of an institution’s TOC processes [3,5]. The Hospital Readmissions Reduction Program implemented by the Centers for Medicare and Medicaid Services (CMS) in 2012 reduced payment to hospitals with excess 30-day readmissions for selected conditions, including chronic obstructive pulmonary disease (COPD) and heart failure (HF). There are also financial incentives provided to acute care facilities that provide quality care under the CMS initiative Hospital Value-Based Purchasing Program [5,6,7,8]. In addition to playing a role in reimbursement, thirty-day readmission rates also often factor into facility quality ratings in the private sector as well as within the Veterans Health Administration (VHA). For example, the Strategic Analytics for Improvement and Learning (SAIL) model was developed by the Department of Veterans Affairs to measure, evaluate, and benchmark quality and efficiency at Veterans Affairs Medicals Centers (VAMC). The SAIL model evaluates 10 measures with one being care transitions, and performance in relation to this model was considered when evaluating and assigning VAMC star ratings, which have been used to compare VA facilities in quality. After implementation of these programs and quality ratings systems, many healthcare facilities turned greater focus to identify interventions to enhance care transitions to improve quality and reduce readmission rates. Despite the vast number of institutions working to identify effective models and interventions to improve TOC, the available literature is limited, conflicting, and describes a variety of models including time of intervention (before, during, or after discharge) and practitioners performing the intervention(s) (multidisciplinary, pharmacist-only, nurse-only) [4,6,7].

As one of the most accessible healthcare professionals, pharmacists have the unique opportunity to provide this care in a timely manner to improve TOC and possibly reduce readmissions. Much of the current literature evaluating pharmacist interventions in transitions of care focuses on pharmacy involvement at discharge, however adverse events and hospital admissions and re-admissions for ambulatory care sensitive conditions (ACSC) such as HF and COPD are considered largely preventable if ambulatory care is provided in a timely and effective manner [8,9]. Within the VHA, the ambulatory care clinical pharmacy specialist (CPS) serves a critical role within the healthcare team. Functioning as a mid-level provider with a scope of practice, the CPS provides comprehensive medication management services to patients with a wide variety of medical conditions. CPSs with a scope of practice, as part of comprehensive medication management, have the ability to perform physical assessment, hold prescriptive authority (which includes initiation, modification, renewal, or discontinuation of medication therapy), order, interpret, and monitor laboratory results, develop patient-centered therapeutic plans, and manage acute and chronic disease states and processes in which medications are the primary treatment. These patient care encounters consist of face-to-face visits in clinic, telephone-based care, or clinical video telehealth. CPSs are able to conduct all components of the medication management through telephone-based care and video telehealth encounters except the physical assessment. This role and accessibility of the CPS within the VHA provides the unique ability and opportunity for the CPS to make impactful interventions for patients, especially during the post-discharge period.

Overall, the literature evaluating the role of the ambulatory care CPS in TOC is limited and the most efficacious model for ambulatory care CPS involvement in TOC remains uncertain. However, improvement in TOC, patient outcomes, and readmission rates through enhancing pharmacist involvement in TOC has been demonstrated within many healthcare facilities including other VHA healthcare systems [4,7,8,10,11,12,13,14]. Key initiatives of these programs included medication reconciliation, pharmacist discharge education, care coordination, and post-discharge follow up with a CPS for discharge diagnosis-specific education and often medication management.

Prior to launching this quality improvement initiative, the healthcare system in which this intervention took place had a number of previously implemented processes in place aimed at ensuring smooth TOC to prevent readmissions including pharmacist medication reconciliation and education at discharge as well as nursing telephone calls to the patient at 2- and 7-day post-discharge. For the time period of 1 January 2018 to 30 June 2018, the average institutional compliance for 2- and 7-day nursing discharge calls were higher than the VHA national average (2-day ~64% and 7-day ~78% vs. 2-day ~62% and 7-day ~72%, respectively). Since the facility was surpassing the national average for nursing discharge calls, it was hypothesized that implementation of a new process enhancing the role of CPS in the post-discharge period could provide additional benefit in order to improve TOC and decrease 30-day readmission rates.

The purpose of this quality improvement project was to evaluate the impact of CPS involvement in the post-discharge period on 30-day readmission rates within a Veterans Affairs Healthcare System. The objectives of this project were to compare the pre- and post-intervention rate of index and all-cause readmissions, identify interventions made by the CPS during the post-discharge period, and describe the impact of early intervention by a CPS on 30-day readmission rates.

## 2. Materials and Methods 

### 2.1. Design

This study followed a prospective cohort design conducted at the Central Alabama Veterans Healthcare System (CAVHCS). CAVHCS is comprised of three main campuses and five community-based outpatient clinics, which altogether serve more than 30,000 Veterans across central Alabama and southwest Georgia. All CAVHCS campuses and clinics participated in this study. The pre-intervention time period was pre-specified as 15 October 2017 through 14 January 2018. The post-intervention time period was 15 October 2018 through 14 January 2019. This project was performed to improve patient care and was determined to meet guidelines for quality improvement exemption not requiring institutional review board review and approval.

### 2.2. Patient Selection

A dashboard was created to promptly identify patients of the facility who were discharged from a VHA acute care facility with a principle or secondary discharge diagnosis of either HF or COPD from 15 October 2018 through 14 January 2019. Patients identified by the dashboard were included in the intervention group unless they met one of the following exclusion criteria: enrolled in the VHA home care program known as home-based primary care which provides primary care services in the homes of homebound patients, discharged to a long-term care or skilled nursing facility, not actively managed by CAVHCS and currently established with another VA healthcare system, discharge diagnosis erroneously coded for HF or COPD, or discharged with hospice or palliative care services.

### 2.3. Interventions

Once a patient was identified by the dashboard and enrolled, a CPS attempted to contact the patient by telephone within 7 days of discharge. This encounter is referred to as the primary visit and focused on medication reconciliation, ensuring continuity of care, and disease state counseling. Medication management was also performed if time permitted and deemed necessary by the CPS conducting this visit. After the primary visit, the secondary visit was scheduled targeting a date within 21 days of discharge. If the CPS was unable to reach the patient by telephone within 7 days of discharge despite at least daily attempts, the primary visit was to be omitted and the CPS targeted follow-up within 21 days post-discharge. The secondary visit was conducted over the telephone or in clinic and focused on medication management. In-clinic visits were preferred for the secondary visit; however, as this facility serves a rural population, if the patient was unable to travel to the clinic within the specified time period, telephone visits were scheduled. Subsequent follow-up with the CPS was scheduled if deemed necessary by the CPS. Standardized note templates for primary and secondary visits were created and used by all 14 CPSs conducting post-discharge visits.

### 2.4. Outcomes

Primary outcomes evaluated were 30-day COPD readmission rate for index diagnosis and 30-day HF readmission rate for index diagnosis. Secondary outcomes were 30-day all-cause readmission rate, average time from discharge to primary visit, average time from discharge to secondary visit, percent of patients who had pharmacological interventions made by CPS, and percent of patients who had non-pharmacological interventions made by CPS. For patients who had a diagnosis of HF with reduced ejection fraction (HFrEF), the percent of patients on appropriate therapy at discharge, by the end of the primary visit, and by the end of the secondary visit were also reported. For the purposes of this study, “appropriate therapy” was defined as sacubitril/valsartan (Entresto) or evidence-based angiotensin converting enzyme inhibitor (ACEI) or angiotensin receptor blocker (ARB) and beta-blocker (BB) as indicated by 2013 ACCF/AHA HF guidelines and the 2017 ACC/AHA/HFSA Focused Update of the 2013 guidelines [15,16].

### 2.5. Data Collection and Statistical Analysis

All baseline and intervention data were obtained from chart review of the electronic medical record conducted by the primary author. Patients who had at least one CPS visit within 30 days of discharge were included in statistical analyses. Chi-squared testing was used to evaluate the change in readmission rates. For all statistical tests, significance was set at *p* < 0.05.

## 3. Results

### 3.1. Cohort Demographics

A total of 46 patients met inclusion criteria and were enrolled in the post-intervention cohort (Table 1). Nine patients (19.6%) were discharged with discharge diagnoses of both COPD and HF. Fifteen patients (32.6%) had a principle or secondary discharge diagnosis of only COPD and 22 patients (47.8%) were discharged with only HF as a principle or secondary discharge diagnosis. The pre-intervention cohort had a total of 96 patients comprised of 19 patients (19.8%) discharged with discharge diagnoses of both COPD and HF, 38 patients (39.6%) having a principle or secondary discharge diagnosis of COPD and 39 patients (40.6%) having a principle or secondary discharge diagnosis of HF. Pre- and post-intervention groups had a similar proportion of males and average age was also similar in both groups (Table 1).

### 3.2. Primary Outcomes

There was a numerical, but non-statistically significant decrease in 30-day index readmissions for both COPD and HF. The pre-intervention 30-day index readmission rate for COPD was 3.51% (2/57) compared to the post-intervention rate of 0% (0/24) (*p* = 0.35). Similarly, the pre-intervention 30-day index readmission rate for HF was 10.34% (6/58) compared to the post-intervention rate of 3.22% (1/31) (*p* = 0.23) (Table 2).

### 3.3. Secondary Outcomes

**Figure 1:**
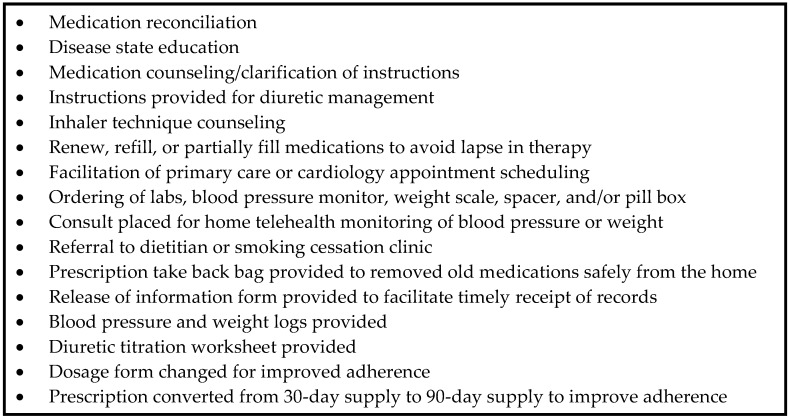
Nonpharmacological interventions made.

There was also a numerical, but non-statistically significant decrease in the 30-day all-cause readmission rate. The rate for the pre-intervention cohort was 11.46% (11/96) and decreased to 8.70% (4/46) (*p* = 0.62) for the post-intervention cohort (Table 2). The average time to primary and secondary visits were 6.2 and 20.6 days, respectively. Forty-three patients (93.5%) had primary visits with an average follow-up time of 6.2 days (3–17) post-discharge. A total of 41 patients (89.1%) received secondary visits with an average follow-up time of 20.6 days (6–43) post-discharge (Table 3). Twenty-one out of 46 patients (45.7%) received at least one pharmacological intervention and 45 out of 46 patients (97.8%) received at least one non-pharmacological intervention. A detailed list of the nonpharmacological interventions made can be found in Figure 1.

**Figure 2:**
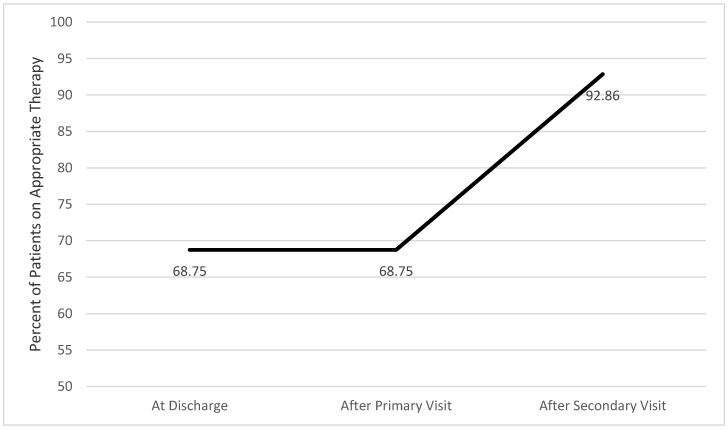
Change in percent of patients with heart failure (HF) with reduced ejection fraction (HFrEF) on appropriate therapy.

Sixty-nine percent of patients (11/16) with HFrEF were receiving appropriate therapy at discharge and after the primary visit was conducted. Fourteen of these patients had a secondary visit as one patient passed away after the primary visit and another did not attend their scheduled secondary visit. After the secondary visit, 13 of the 14 patients (93%) were determined to be receiving appropriate therapy, representing an increase of 24% (Figure 2).

## 4. Discussion

As part of this quality improvement project, CPS involvement in the facility’s TOC process for patients with a discharge diagnosis of HF or COPD was enhanced by implementing two post-discharge visits with a CPS. A total of 46 patients received at least 1 visit with a CPS within 30 days of their discharge from a VA acute care facility with a principle or secondary diagnoses of COPD or HF. Pre- and post-intervention groups had a similar proportion of males and average age was also similar in both groups, however the post-intervention cohort (*n* = 46) was significantly smaller than the pre-intervention cohort (*n* = 96). The reason for this disparity was that the dashboard identification system was able to identify in real-time some, but not all patients who were hospitalized at a VA acute care facility outside of the Central Alabama Veterans Healthcare System. These delays resulted in many patients who would have met inclusion criteria being identified after 30 days had already elapsed since the index hospital discharge.

There was a numerical decrease in 30-day index and all-cause readmissions for both COPD and HF, however these decreases did not reach statistical significance. The average time to primary and secondary visits both met the pre-specified targets of within 7 and 21 days post-discharge, respectively, however the range for both was relatively wide, including some patients with a secondary visit after 30 days post-discharge. The timing of primary and secondary visits proved to be an unforeseen barrier, limitation, and possible confounder in this study. All 3 patients with a secondary visit occurring after 30 days post-discharge did have a primary visit relatively soon after discharge (7, 8, and 9 days). Eight of the included 46 patients only received one encounter with a CPS, 3 with a primary visit only, and 5 with a secondary visit only. As mentioned earlier, if a CPS was unable to contact the patient within 7 days of hospital discharge, the primary visit was to be omitted and only a secondary visit was to be conducted. However, due to the many holidays and staffing shortages encountered during the pre-specified time period (15 October 2018 through 14 January 2019), some delays in identification of patients by the dashboard, and patients returning calls left by the CPS after 7 days post-discharge, some patients who made contact after 7 days post-discharge received a primary and secondary visit, explaining the wide range for time to primary visit. Other barriers to conducting visits within the pre-specified target date range were CPS clinic availability, inability to reach patients by telephone initially or for scheduled telecare follow ups, and delay in scheduling CPS visits by support staff. Only including patients who had both a primary visit within 7 days and secondary visit within 21 days could have been beneficial to evaluate the impact of the follow-up model used, however it was felt by the investigators that including all patients with at least one CPS encounter within 30 days of discharge would improve external validity and better evaluate the benefit of continuing the service at this facility.

Almost half of included patients received a pharmacologic intervention made by a CPS during the post-discharge time period and all but one received a non-pharmacologic intervention. This shows that the follow up encounters with the CPS were productive and likely impacted the results seen. In addition to using the scope of practice to make pharmacologic interventions, there was a wide array of non-pharmacologic interventions made that may have reinforced the discharge counseling the patient received from inpatient nursing or pharmacy staff, but also may have been original interventions including preventing lapses in medications and referrals to other services or specialists.

The percent of patients with HFrEF on appropriate therapy was stable at 69% from discharge to the end of the primary visit and increased to 93% which correlates to all but 1 patient being prescribed appropriate therapy by the end of the secondary visit. These results possibly allude to 2 post-discharge visits being optimal for those patients discharged with a diagnosis of HFrEF.

Power was not calculated; however, it is possible that the sample size was not large enough to see a statistically significant difference. As stated earlier, however, this was a quality improvement project, and the decrease in readmissions could be very impactful for the facility and patient care, especially considering the absence of COPD index readmissions during the post-intervention period compared to the rate of over 5% in the pre-intervention period. It is also likely that this service could provide benefit and clinical significance if this service were expanded to encompass patients admitted for other ambulatory care sensitive conditions in addition to the two targeted by this service, HF and COPD.

In addition to the limitations listed prior, one additional confounding factor to note is that at the time of initiation of this service, the facility also launched a separate initiative to improve care and outcomes for patients admitted for HF exacerbations. This included reserving specific clinic days for cardiologists to see HF patients who were recently discharged with an exacerbation. The number of the patients included in pre- and post-intervention cohort who were seen by a cardiologist within 30 days of discharge was not collected. However, it is likely this service also faced some of the same scheduling barriers that affected the CPS and the cardiology service was not using any identification system to identify patients discharged from other VHA facilities.

## 5. Conclusions

CPS involvement in the post-discharge period reduced index and all-cause readmission rates for patients discharged with a principle or secondary discharge diagnosis of COPD or HF. In regards to model, two post-discharge visits with a CPS within 30 days of hospital discharge may be a beneficial structure for CPS intervention during the post-discharge period, especially for those patients with a diagnosis of HFrEF.

## Figures and Tables

**Table 1:** Baseline demographics and characteristics by cohort.

	Pre-Intervention (*n* = 96)	Post-Intervention (*n* = 46)
**COPD only**	38 (39.6%)	15 (32.6%)
**HF only**	39 (40.6%)	22 (47.8%)
**COPD + HF**	19 (19.8%)	9 (19.6%)
**Male**	92 (95.8%)	44 (95.7%)
**Age (mean, SD)**	67.8 (9.8)	67.0 (9.3)

**Table 2:** Readmission rates.

	Pre-Intervention % (#/n)	Post-Intervention % (#/n)	*p*
**30-day index**			
COPD	3.51 (2/57)	0 (0/24)	0.35
HF	10.34 (6/58)	3.22 (1/31)	0.23
**30-day all-cause**	11.46 (11/96)	8.70 (4/46)	0.62

**Table 3:** Secondary outcomes for all patients.

Average Time to Follow-Up (Range)	
Primary visit (*n* = 43)	6.2 days (3–17)
Secondary visit (*n* = 41)	20.6 days (6–43)
**Percent of Patients with Intervention Made by CPS**	
Pharmacologic interventions	45.7% (21/46)
Non-pharmacologic interventions	97.8% (45/46)
